# Supplementary material for: OxPhos defects cause hypermetabolism and reduce lifespan in cells and in patients with mitochondrial diseases
Source: Commun Biol. 2023 Jan 12;6:22. doi: 10.1038/s42003-022-04303-x (PMC9837150; doi:10.1038/s42003-022-04303-x)
Supplement: Supplementary file 3 — Description of Additional Supplementary Files [file 42003_2022_4303_MOESM3_ESM.pdf]

Description of Additional Supplementary Files

File name: Supplementary Data 1
Description: Table of differentially expressed genes for SURF1 cells over 0 to 75 days of growth.

File name: Supplementary Data 2
Description: Table of differentially expressed genes for Oligo cells over 35 to 110 days of growth.

File name: Supplementary Data 3
Description: Table of differentially expressed genes shared across SURF1 and Oligo cells

File name: Supplementary Data 4
Description: Differentially methylated CpGs for SURF1 cells over 0 to 75 days of growth.

File name: Supplementary Data 5
Description: Differentially methylated CpGs for Oligo cells over 35 to 110 days of growth.

File name: Supplementary Data 6
Description: Differentially methylated CpGs shared across SURF1 and Oligo cells.

File name: Supplementary Data 7
Description: Differentially methylated regions for SURF1 cells over 0 to 75 days of growth.

File name: Supplementary Data 8
Description: Differentially methylated regions for Oligo cells over 35 to 110 days of growth.

File name: Supplementary Data 9
Description: Differentially methylated regions shared in both SURF1 and Oligo cells.
