# Supplementary material for: OxPhos defects cause hypermetabolism and reduce lifespan in cells and in patients with mitochondrial diseases
Source: Commun Biol. 2023 Jan 12;6:22. doi: 10.1038/s42003-022-04303-x (PMC9837150; doi:10.1038/s42003-022-04303-x)
Supplement: Supplementary file 13 — Reporting Summary [file 42003_2022_4303_MOESM13_ESM.pdf]

nature portfolio

Corresponding author(s): Martin Picard

Last updated by author(s): Oct 21, 2022

Reporting Summary

Nature Portfolio wishes to improve the reproducibility of the work that we publish. This form provides structure for consistency and transparency in reporting. For further information on Nature Portfolio policies, see our Editorial Policies and the Editorial Policy Checklist.

Statistics

For all statistical analyses, confirm that the following items are present in the figure legend, table legend, main text, or Methods section.

n/a	Confirmed	
☐	☒	The exact sample size (n) for each experimental group/condition, given as a discrete number and unit of measurement
☐	☒	A statement on whether measurements were taken from distinct samples or whether the same sample was measured repeatedly
☐	☒	The statistical test(s) used AND whether they are one- or two-sided<br>*Only common tests should be described solely by name; describe more complex techniques in the Methods section.*
☐	☒	A description of all covariates tested
☐	☒	A description of any assumptions or corrections, such as tests of normality and adjustment for multiple comparisons
☐	☒	A full description of the statistical parameters including central tendency (e.g. means) or other basic estimates (e.g. regression coefficient) AND variation (e.g. standard deviation) or associated estimates of uncertainty (e.g. confidence intervals)
☐	☒	For null hypothesis testing, the test statistic (e.g. F, t, r) with confidence intervals, effect sizes, degrees of freedom and P value noted<br>*Give P values as exact values whenever suitable.*
☒	☐	For Bayesian analysis, information on the choice of priors and Markov chain Monte Carlo settings
☒	☐	For hierarchical and complex designs, identification of the appropriate level for tests and full reporting of outcomes
☐	☒	Estimates of effect sizes (e.g. Cohen's d, Pearson's r), indicating how they were calculated

Our web collection on statistics for biologists contains articles on many of the points above.

Software and code

Policy information about availability of computer code

Data collection	Data collection software includes affiliated instrument software including Seahorse XF analyzer, QuantStudio 7 flex qPCR, and Luminex 200.
Data analysis	Data analysis software includes : GraphPad Prism (v9.0), RStudio (v1.3.1056) using R (v4.0.2) and packages ‘ggplot2’(v3.3.5), ‘Rtsne’ (v0.15) , ‘lme4’ v1.1, ‘minfi’ (v1.36.0), ‘sva’ (v3.12.0), 'kallisto' (v0.44.0), ‘tximport’ (v1.18.0), ‘DEseq2’ (v1.30.1), as well as computational pipelines eKLIPse (Goudenège et al, GIM, 2019; https://github.com/dooguypapua/eKLIPse), iPAGE (https://tavazoielab.c2b2.columbia.edu/iPAGE/), DNAmAge online calculator (https://dnamage.genetics.ucla.edu/new), and PC-based DNAmAges calculator (https://github.com/MorganLevineLab/PC-Clocks).<br><br>An analysis software was developed using the ShinyGo (v0.66) framework. The designed webtool enables multimodal exploration of this longitudinal dataset and is publicly available at: https://columbia-picard.shinyapps.io/shinyapp-Lifespan_Study/

For manuscripts utilizing custom algorithms or software that are central to the research but not yet described in published literature, software must be made available to editors and reviewers. We strongly encourage code deposition in a community repository (e.g. GitHub). See the Nature Portfolio guidelines for submitting code & software for further information.

Data

Policy information about availability of data

All manuscripts must include a data availability statement. This statement should provide the following information, where applicable:

- Accession codes, unique identifiers, or web links for publicly available datasets
- A description of any restrictions on data availability
- For clinical datasets or third party data, please ensure that the statement adheres to our policy

The meta-analyzed clinical data of mitochondrial disease cohorts (Figure 1) can be obtained from the original publications listed in Table 1. The complete fibroblast dataset for the present study is available without restriction and can be accessed, visualized, and downloaded using our webtool: https://columbia-picard.shinyapps.io/shinyapp-Lifespan_Study/. Data presented in this manuscript was generated as part of the Cellular Lifespan Study, which includes metabolic and endocrine experimental treatments across multiple donors described in detail in Refeference 109. The RNAseq and DNA methylation datasets for this project are available under the GEO SuperSeries GSE179849. All data preprocessing and analysis code is available on GitHub (https://github.com/gav-sturm/Cellular_Lifespan_Study).

Field-specific reporting

Please select the one below that is the best fit for your research. If you are not sure, read the appropriate sections before making your selection.

☒ Life sciences ☐ Behavioural & social sciences ☐ Ecological, evolutionary & environmental sciences

For a reference copy of the document with all sections, see nature.com/documents/nr-reporting-summary-flat.pdf

Life sciences study design

All studies must disclose on these points even when the disclosure is negative.

Sample size	Clinical data was aggregated from previously published data spanning 17 cohorts and totaling 225 healthy controls and 690 patients. All cellular lifespan experiments were performed with n=3 controls and n=3 patient or treatments groups. Donors were selected to ensure representation of both male and female sexes and that donors were <20 years of age.
Data exclusions	A fourth patient-derived fibroblast cell line underwent cell death within 14 days of culturing and was unable to be included in the current study.
Replication	Cellular lifespan experiments were replicated in two rounds of the study performed by different investigators. Further, longitudunally sampling bioenergetic and molecular parameters across cellular lifespan ensures that stable and time-independent experimental effects for each individual measure are obtained. All Seahorse assays were run with 10-12 technical replicates, qPCR measures were run in technical triplicates, cytokine measures were run in technical duplicates. Telomere length, DNA methylation, and RNAsequencing analyses were performed once on each sample, as described in the methods.
Randomization	For large-batch measurements (>100 samples), samples were randomized across plates to remove order or plate affect. All samples were further encoded to ensure technician was blinded to sample identity during processing and measurement.
Blinding	Investigators were not blinded during sample collection due to the nature of the long-term longitudinal cellular lifespan study design. Each cell line grows at its own rate and requires biweekly maintenance and passaging making it technically challenging to anonymous cell line identity while ensuring matching collection points and equal distribution of control and experimental samples.

Reporting for specific materials, systems and methods

We require information from authors about some types of materials, experimental systems and methods used in many studies. Here, indicate whether each material, system or method listed is relevant to your study. If you are not sure if a list item applies to your research, read the appropriate section before selecting a response.

Materials & experimental systems

n/a	Involved in the study	
☒	☐	Antibodies
☐	☒	Eukaryotic cell lines
☒	☐	Palaeontology and archaeology
☒	☐	Animals and other organisms
☐	☒	Human research participants
☒	☐	Clinical data
☒	☐	Dual use research of concern

Methods

n/a	Involved in the study	
☒	☐	ChIP-seq
☒	☐	Flow cytometry
☒	☐	MRI-based neuroimaging

Eukaryotic cell lines

Policy information about cell lines

Cell line source(s)	Healthy control fibroblasts were sourced from Lifeline Cell Technology or Coriell Institute. Patient-derived fibroblasts were collected from local-clinic and processed in-house. See Extended table 1 for detailed breakdown of cell line source.
Authentication	The sex of every cell line was validated using whole-genome sequencing and DNA methylation arrays. The clinical genotype of patient-derived fibroblasts was validated with whole-genome sequencing.
Mycoplasma contamination	Mycoplasma testing was performed according to the manufacturer's instructions (R&D Systems #CUL001B) at the end of lifespan for each treatment and cell line used. All tests were negative.
Commonly misidentified lines (See ICLAC register)	*Name any commonly misidentified cell lines used in the study and provide a rationale for their use.*

Human research participants

Policy information about studies involving human research participants

Population characteristics	The physiological data in Figure 1 includes healthy participants and patients with various mitochondrial diseases including MERRF, KSS, CPEP, MELAS, SURF1, and LRPPRC (see Table 1 for details). Participants ranged 0-100 years of age, spanning diverse geographical and ethnic populations. All data was previously published with each cohort containing its own population of interest, unless otherwise noted in the materials and methods.<br><br>Primary human dermal fibroblasts were obtained from 3 patients carrying a mutation in the SURF1 gene. Extended table 1-2 detail donor characteristics and genotype.
Recruitment	Patient-derived fibroblasts were obtained from local-clinic with informed consent.
Ethics oversight	Columbia Medical Center IRB #AAAB0483

Note that full information on the approval of the study protocol must also be provided in the manuscript.
